# The Interaction between Selection, Demography and Selfing and How It Affects Population Viability

**DOI:** 10.1371/journal.pone.0086125

**Published:** 2014-01-21

**Authors:** Diala Abu Awad, Sophie Gallina, Cyrille Bonamy, Sylvain Billiard

**Affiliations:** 1 UMR-CNRS 8198, Laboratoire Génétique et Évolution des Populations Végétales, Université Lille 1, Villeneuve d'Ascq, France; 2 Centre de Ressources Informatiques (CRI), Université Lille 1, Villeneuve d'Ascq, France; Centers for Disease Control and Prevention, United States of America

## Abstract

Population extinction due to the accumulation of deleterious mutations has only been considered to occur at small population sizes, large sexual populations being expected to efficiently purge these mutations. However, little is known about how the mutation load generated by segregating mutations affects population size and, eventually, population extinction. We propose a simple analytical model that takes into account both the demographic and genetic evolution of populations, linking population size, density dependence, the mutation load, and self-fertilisation. Analytical predictions were found to be relatively good predictors of population size and probability of population viability when verified using an explicit individual based stochastic model. We show that initially large populations do not always reach mutation-selection balance and can go extinct due to the accumulation of segregating deleterious mutations. Population survival depends not only on the relative fitness and demographic stochasticity, but also on the interaction between the two. When deleterious mutations are recessive, self-fertilisation affects viability non-monotonically and genomic cold-spots could favour the viability of outcrossing populations.

## Introduction

Population size and viability are both affected by extrinsic (e.g. environmental change and interspecific interactions) and intrinsic factors (e.g. genetic and demographic components). The genetic factors most frequently considered as contributing to population decline are the lack of adaptive potential in a changing environment, inbreeding depression and the reduction of fitness due to the accumulation of deleterious mutations (reviewed in [1]). The accumulation of deleterious mutations has often been put forth as an explanation for species extinction, especially concerning the differences in extinction rates between sexual and asexual species, or selfers and outcrossers. The relevance of the accumulation of deleterious mutations on population extinction, however, remains unclear.

Both empirical and theoretical works have insisted on the importance of deleterious mutation fixation on the decline and extinction of populations. Some models have shown that small populations can go extinct due to the acceleration of recurrent fixation of deleterious mutations with a small effect, the so called mutational meltdown [2]–[5]. Several empirical works have also supported this hypothesis. The fitness of experimental populations has been shown to decrease after several generations during which new deleterious mutations are fixed [6]–[10], and data from small highly inbred natural populations follow the same trend [11]–[13]. However, in these theoretical and empirical works, populations are considered to be small and isolated or, because of successive growth-dilution cycles, are subjected to recurrent and strong bottlenecks. When these conditions are not met (i.e. when populations are larger, or are not isolated or are not subject to strong recurrent bottlenecks) populations are more likely to go extinct because of other genetic and demographic factors before the fixation of deleterious mutations has an effect on population decline [1].

What about large populations, can they decline in size due to recurrent deleterious mutations? It has been suggested that the mutation load due to segregating mutations might be important in population decline [4], [14]. This however remains controversial as it is generally thought that in large sexual populations deleterious mutations should be efficiently purged [15], [16]. First, segregating deleterious mutations are expected to have no consequence on demography [17], especially in the presence of density-dependence, where there is compensation of the death of individuals due to selection by those that would have been lost from the population due to the lack of resources (soft selection). Empirical evidence on the other hand supports more “hard selection” [18], in which density-independent deaths due to the genetic load are not completely compensated. It is crucial to determine whether segregating mutations are important or not for determining population viability as this has direct empirical implications, especially when considering the genetic rescue of populations. Second, many aspects of population survival and extinction in presence of a high mutation load still remain unclear. When taking into account empirical estimations of genomic mutation rates (between 0.1 and more than 1 for higher Eukaryotes per genome and per generation, [19]) and the effects of deleterious mutations, population genetics theories [20] imply that we should expect extremely high mutation loads. Population genetics models consider population size as a fixed variable, and their stochastic estimations of the mutation load, even in finite populations, also agree with the existence of high mutation loads [21], [22]. Can these predictions still hold if we allow selection to influence population size, or would the mutational load evolve differently? One of the earliest models, to our knowledge, that has taken into account the effect of the mutation load on mean fitness, and the latters effect on population size is a single-locus model proposed by Clarke [23]. In this model, the mutation load had a very small, almost negligible effect on population size, however Clarke [23] verbally suggested that extending the model to a whole genome could possibly lead to a more important effect.

In order to better understand the interaction between the mutation load and demography, we propose a model that combines simple deterministic population genetics and demographic models. We consider sexual reproduction, with a mean reproductive rate that depends both on population density and on the population's mutation load, and recurrent mutations segregating at an infinite number of loci. Using this model we predict the relationship between population size and the mutational load at mutation-selection equilibrium. We also predict the threshold fitness value depending on both the genetic and demographic parameters under which the population is not expected to be viable. These predictions are then verified using an individual-based simulation model in which we explicitly model the introduction of new mutations in the genome and the effect of the mean genomic recombination rate. Population size varies from one generation to the next, as it depends on individual fitness and competition. This simulation model allows for the better understanding of the mechanisms leading to population extinction, more specifically the relative importance of the polymorphism of deleterious mutations, the fixation of these mutations, and the mutational meltdown.

To illustrate the importance of such an approach (i.e. combining genetic and demographic factors) in ecology and evolution, we will address the question of the effect of self-fertilisation on population size and viability. This is indeed a long running question, as the prevalence of outcrossing species is still puzzling both in animals and plants [24]. From an evolutionary standpoint, self-fertilisation should be greatly advantaged because of Fisher's transmission advantage [25], a more efficient purge of deleterious mutations [26], and also because of their reproductive assurance (e.g. Baker's law, [27], [28]). This is correlated with the empirical estimation of high transition rates from outcrossing to selfing reproductive systems, for instance in the Solanaceae [29]. Despite this transition rate, and high speciation rates in selfers compared to outcrossers, outcrossers still represent more than 40% of species in this family. Other studies in other plant families also come to this conclusion [30]–[32]. Goldberg *et al.* [29] show that this puzzling prevalence of outcrossers is due to higher extinction rates in selfing species than in outcrossing ones. One hypothesis to explain this difference in extinction rates is that selfers are more prone to mutational meltdown than outcrossers [4]. Empirical work on fungi, more specifically *Neurospora*, strongly supports this hypothesis, as selfing lineages accumulate more deleterious mutations and are less fit than outcrossing ones [33]. We will therefore extend our model in order to include different rates of self-fertilisation and test this hypothesis.

## Model

### Deterministic model and expectations

We consider a population in a constant environment, with discrete, non-overlapping generations. At generation *t*, the population is made up of 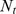 hermaphrodite individuals, where
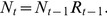
(1)

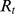
 is the absolute multiplicative fitness of a population at a given generation 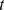, with trait value (or relative fitness) 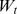, in a population of density 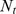 [34] is given by
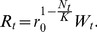
(2)


The density-dependent component of fitness depends on 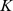, the carrying capacity of a population with all individuals having the optimal relative fitness, and on 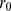, the intrinsic reproductive rate of such a population [34]. The second factor, 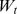, corresponds to the mean relative fitness of individuals in the population, as a function of their mutation load, and so on the number of segregating or fixed deleterious mutations in the population. In this model, we consider that density dependence affects all individuals in the same way, independently of their relative fitness (or genotype).

If the mean relative fitness is at equilibrium (when the population is at mutation-selection balance), and there is no demographic stochasticity, the equilibrium of population size denoted 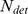, can be expressed as a function of the relative fitness at equilibrium 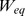, 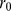 and 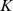, giving
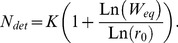
(3)


By solving 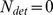 from equation 3, we can determine the relative fitness threshold
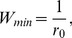
(4)under which the population is not expected to be viable.

A deterministic value of 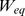, noted 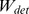, can be calculated at mutation-selection balance for a large population of diploid individuals with a large number of independent bi-allelic loci, where deleterious mutations with selection coefficient 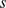 and dominance 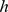 can segregate. This is done using equations for the mutation load 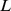 derived from Wright's equations for allele frequencies at equilibrium at a single locus [35], [36], and gives
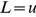
(5a)for recessive mutations (the dominance coefficient 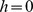), and
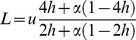
(5b)when 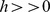, where 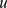 is the deleterious mutation rate at a single locus and 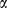 is the proportion of offspring produced via self-fertilisation. If we consider that there is no epistasis and no linkage disequilibrium, then the relative population fitness when considering a genome-wide mutation load is given by 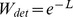 [20], where 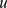 is replaced by 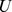, the haploid genomic mutation rate, when calculating 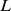.

Finally we can calculate 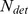, using 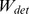 instead of 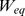 in Equation 3 as an estimation of population size at equilibrium 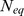. Populations are not expected to be viable (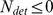) when for 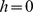


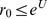
(6a)and for 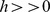


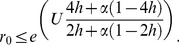
(6b)


High mutation rates and low reproductive rates are both expected to contribute to the decrease of population viability.

### Simulation model

An individual-centred model with discrete non-overlapping generations was used to follow the evolution of an isolated population of variable size, made up of diploid hermaphrodite individuals.

#### Genomic assumptions

The genetic properties of this model, mutation and recombination, are those described in Roze's [37] model. Each individual is represented by two homologous chromosomes of length 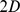 with a potentially infinite number of loci. The map length is considered to be 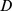 from the centre of the chromosome to the edge, hence representing a chromosome with a defined centromere. The life cycle is as follows: mutation, selection, meiosis and reproduction.

The number of new deleterious mutations occurring per chromosome per generation, is sampled from a Poisson distribution with mean 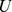, where 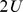 is the genomic mutation rate. Their position on the chromosome is sampled from a uniform distribution in 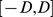. The effect of deleterious mutations on the fitness of individual 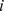 living at time 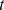, 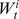, is multiplicative and depends on the number of homozygous, 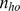, and heterozygous, 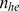, deleterious mutations each individual carries
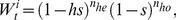
(7)where 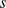 and 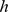 are the selective and dominance coefficients respectively and are fixed parameters. All mutations are deleterious and have the same values of 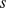 and 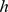. The deleterious effect of these mutations is independent of population density. Recombination occurs during gamete production and is considered to be uniform along the chromosome. New individuals are a combination of two gametes, either from two different individuals for reproduction via outcrossing, or the same individual via selfing.

#### Demography and selection

At a given time 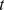, population size 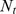 is given by
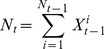
(8)where 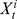 is the number of viable offspring an individual 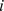 at time 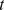 contributes to the next generation via the female function (we consider that there is no limitation in the number of offspring an individual 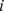 can sire). 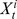 is sampled from a Poisson distribution with mean 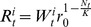 (the individual reproductive rate).

Self-fertilisation occurs at a probability 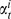 for individual 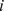, at time 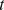 given by
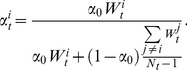
(9)


The proportion of selfed offspring depends on 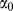, the proportion of an individual's male gametes that are available for self-fertilization, and on the individual's fitness 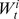 compared to the average relative fitness of the other possible fathers in the population (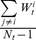). The lower an individual's relative fitness as a father, the lower the proportion of offspring produced via selfing. We consider that there is no limitation in the availability of male gametes. The proportion of an individual's offspring produced by self-fertilisation is sampled from a binomial distribution with parameters 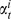 and 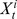. When 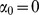 the population is strictly outcrossing and the population is automatically considered non-viable if 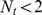.

In order to facilitate the reading of the following sections, all the notations used through the text have been summarized in Table 1.

**Table 1:** Notations.

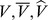	No superscript indicates deterministic values (except in the case of population size 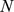 , where it is mentioned clearly in the text), a bar indicates that the variable is the intra-population mean for one simulation run ( 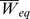 is the mean relative fitness for one population) and a hat indicates that the variable is the mean across all simulations ( 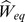 is the mean relative fitness across all simulations, conditional to survival).
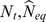	Population sizes at generation 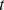 and mean population size at population equilibrium across all simulations
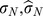	Standard deviation of population size over time as a measure of the fluctuation of population size for one population and across simulations.
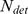	Expected population size without demographic or genetic stochasticity
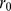	The intrinsic reproductive rate.
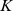	The carrying capacity.
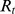	Expected number of offspring per individual at generation 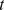 .
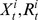	Respectively the number of offspring produced by individual 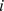 at generation 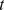 and its expectation.
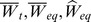	Means of the population's relative fitness at generation 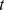 and at population equilibrium for one or across simulations conditional to population survival.
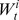	Relative fitness of individual 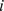 at generation 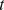 .
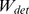	Expected value of the population's component of fitness calculated using the Wright-Fisher model.
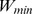	The threshold value of the population's mean relative fitness, under which populations are not viable.
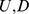	Genomic properties: the haploid mutation rate and the recombination rate or map length.
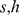	Mutational effects: the selection coefficient and the dominance.
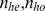	The number of heterozygote and homozygote mutations on an individual's genome.
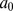	The proportion of male gametes available for selfing.
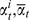	Proportion of offspring produced via self-fertilisation by individual 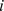 and the population's mean proportion of offspring produced via self-fertilisation at generation 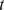 .

#### Initial conditions and simulations run

At the beginning of each simulation, we consider population size to be equal to 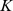 and that there are no deleterious mutations present in the population. The simulations are run until the population reaches equilibrium or goes extinct. We define equilibrium as the stabilisation of the mean population fitness, denoted 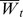; the average 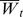 over one thousand generations is calculated and compared to the average 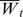 of the previous thousand generations. If the difference between the two is lower than 1 per cent the population is considered to be at equilibrium. Population size 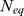, mean fitness 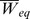 and the mean number of mutations per chromosome were measured at equilibrium. Throughout the results, we will mostly be addressing the average of the mean value of the population's relative fitness across all simulations for each set of parameter values, which we note 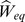. If the population goes extinct, then the time to extinction is measured.

The mutational meltdown is defined as the acceleration of the decrease of population size due to the accumulation of deleterious mutations [3]–[5]. In order to evaluate this acceleration, once non-viable populations reach a population size of 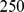 individuals, the best fitting quadratic polynomial regression (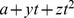) is calculated to fit the decrease in 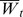, 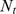 and 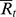 independently of one another. When these variables are decreasing, the first order coefficient is negative. As these variables decrease with time, if they decrease in a linear fashion, then there is no mutational meltdown and the second order coefficient 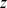 is equal to 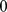. In the case of acceleration of the decrease of these variables with time, as expected in the case of a mutational meltdown, then the second order coefficient 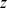, like the first order coefficient 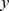, is negative. This second order coefficient 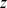 is calculated for each simulation run that results in population extinction. We also measure the mean population size at the fixation of the first deleterious mutation in order to detect whether population decline is associated to mutation fixation.

A wide range of values, from 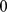 to 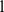, were run for the parameter 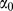, with 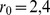 and 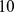. For 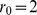, simulations were run for 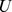 between 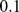 and 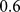, mutations were mildly deleterious, moderately deleterious or lethal (*s* = 0.02, 0.2 and 1), that were completely recessive, almost recessive, or moderately recessive (*h* = 0, 0.02 and 0.2). The recombination rates taken into account reflect conditions where mutations were highly linked, moderately linked or very slightly linked (*D* = 0.1, 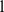 and 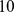). Aside from the general effect of recombination rates over a whole genome, these genomic recombination rates can also reflect how mutation loads evolve within a genome in specific genomic hot and cold spots. Increasing 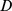 over 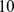 has very little effect on the results, which allows us to make the assumption that the mutations act as though they were independent [38]. However, it is possible, when there is selfing, that there is some linkage due to the genomic consequences of self-fertilisation. One thousand replicates were run for each group of parameter values, coming to a total of 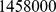 simulations run for 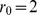. For 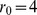 and 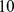, one hundred replicas were run for 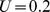 to 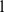, 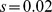 and 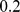, 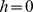 and 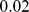 and 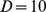, leading to a total of 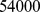 simulations run.

We compare the expected deterministic values of the mean fitness at equilibrium 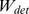, as well as the expected deterministic fitness threshold value 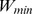 under which populations should not be viable, with our simulation results.

### Estimating the stochastic fluctuations of population size

The stochastic fluctuation of population size from one generation to the next can be due to two mechanisms: demographic stochasticity alone or the interaction between demography and genetic selection. In order to estimate the importance of each of these two sources of stochasticity, we first estimated the fluctuations that would be observed with only demographic stochasticity and no mutations (the relative fitness 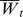 is a constant) and then compared these estimations with the fluctuations observed in our simulations. We use the standard deviation of population size over time 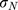 as a measure of these fluctuations. We compare 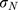 calculated from simulations run for 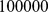 generations for different constant values of 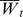 to the standard deviation of 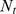 over 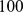 generations when the populations in our simulations were at equilibrium. If demographic stochasticity alone can explain the fluctuation of population size, then the stochastic fluctuations calculated from the simulations with a dynamic component of relative fitness (denoted 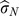) should not be very different than those run with 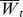 as a constant.

In our simulations, population extinction is inevitable, as when time is very long, all populations go extinct due to demographic stochasticity with a probability of 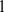. In order to test whether populations that are expected to be viable, in other words with an expected relative fitness 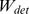 greater than the threshold value 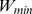 (Equation 3), go extinct because of demographic stochasticity alone, we ran stochastic simulations of Equation 8. We assume that all individuals have the same constant relative fitness 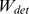 (equation 5) and the initial population size is 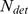 (Equation 3). We calculate the probability of population extinction before one order of magnitude higher than the highest time to extinction observed in our simulations with a dynamic component of relative fitness.

## Results

### Demographic and genetic evolution to equilibrium and extinction

**Figure 1:**
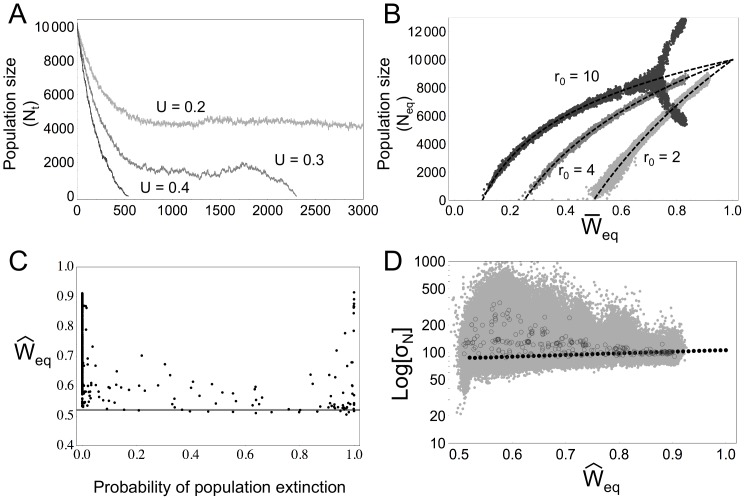
Demographic and genetic evolution of populations. **A**) Typical evolution of population size 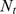 with time for a viable population (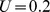), one that reaches equilibrium but goes extinct due to stochasticity (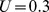), and one that is not viable (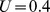). 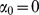, 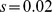, 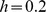, 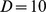, 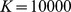 and 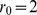. 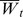 follows the same pattern. **B**) Population size at equilibrium, 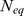 as a function of mean population fitness 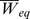 for different values of 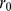. The dashed line represents the expected population size 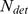 from Equation 3, and the points represent simulation results for all viable populations for all parameter sets with 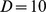 and 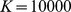. **C**) Probability of population extinction from simulations run for all sets of parameter values for 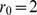 and 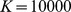 as a function of the average population's mean relative fitness 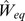. The grey line represents the population fitness above which the probability of population extinction in less than 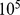 generations due to demographic stochasticity alone is almost null. **D**) Standard deviation of population size over time at population equilibrium 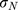 as a function of the relative fitness 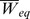 from simulations run for all sets of parameter values for 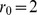 and 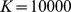. The light grey points each represent 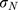 of a single simulation, the open circles represent the mean standard deviation across simulations per group of parameter values 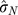 and the full circles represent results from simulations run that take into account only demographic stochasticity.

Populations either evolve both demographically and genetically to a quasi-stationary equilibrium of population size and the relative fitness, noted respectively 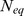 and 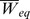, or go extinct (illustrated in Figure 1A).

For all values of the intrinsic reproductive rate 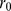, the simulation results agree with deterministic expectations of the interaction between 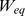 and 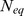 from Equation 3 (see Figure 1B) for viable populations. When 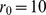 and 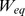 values are greater than 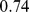, the deterministic equilibrium is oscillatory and unstable, as confirmed by a stability analysis of equation 3. This is clearly seen in the simulation results, where the populations oscillate between two states.

Very few populations are viable with a relative fitness at equilibrium 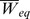 that is lower than the estimated value of the relative fitness threshold 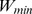, and as expected from Equation 4, 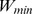 decreases with an increasing intrinsic reproductive rate 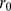. This strong relationship between 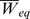 and 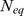 is not enough, however, to explain population extinction. When taking a closer look at the relationship between the probability of population extinction and mean population fitness, we do not have a positive linear relationship between the two variables, but a more bimodal distribution (Figure 1C). We observe a great range of values of 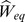 (i.e. the average 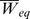 of viable populations) across simulations for which all populations survive, a similar range of 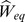 for parameter sets for which very few populations were viable and intermediate-low values of 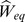 for populations with an intermediate probability of extinction.

When the deterministic value of mean fitness 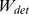 is greater than the fitness threshold 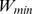 but populations are not viable, extinction can be attributed to either demographic stochasticity alone, or to an interaction between both demographic and genetic stochastic processes. When we consider the mean relative fitness 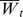 to be a constant, so that population extinction is due only to demographic stochasticity, we find that the probability of extinction is extremely low compared to what is observed in our results. For example for 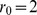 and 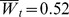, the probability of extinction within a time equivalent to one order of magnitude greater than our highest time to extinction observed is almost null. In other words, these populations are expected to survive as their relative fitness is above the threshold value 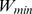, and the expected time to stochastic demographic extinction is extremely long. This however is not the case in simulations with a dynamic component of fitness, suggesting that it is the interaction between demography and genetics that leads to population extinction in such a relatively short time scale.

The importance of this interaction is observed when taking into account the standard deviation of population size over time 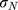 (Figure 1D). We observe that the values of 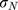 for each simulation run (grey points) fluctuate around the expected standard deviation if the change in population size from one generation to the next were due only to demographic stochasticity (full circles). Fluctuations are even higher when the relative fitness 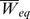 decreases. We note that the mean value of the standard deviation of population size over time 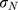 for any group of parameter values 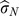 (open circles) is always greater than the expected values of standard deviation of this variable when only demographic stochasticity affects population size. This suggests that the interaction between demography, genetics and selection highly increases the stochastic fluctuations of population size. Below, we show that this increased stochasticity is important when estimating the probability of population extinction.

From this point onwards, we will mostly be addressing results with an intrinsic reproductive rate 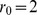 since that we observe the same patterns for higher values of 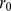 when considering population extinction and mutational loads. For simplicity, in order to compare the effect of selfing between populations, we have chosen to compare population characteristics for different values of the proportion of male gametes available for selfing 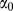 and not 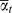, the effective mean proportion of offspring produced via self-fertilisation of the population. The difference between the two is slight (unless the population is very near extinction), with 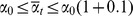, except for 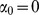 and 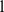, where it remains fixed.

### Probability of population extinction

**Figure 2:**
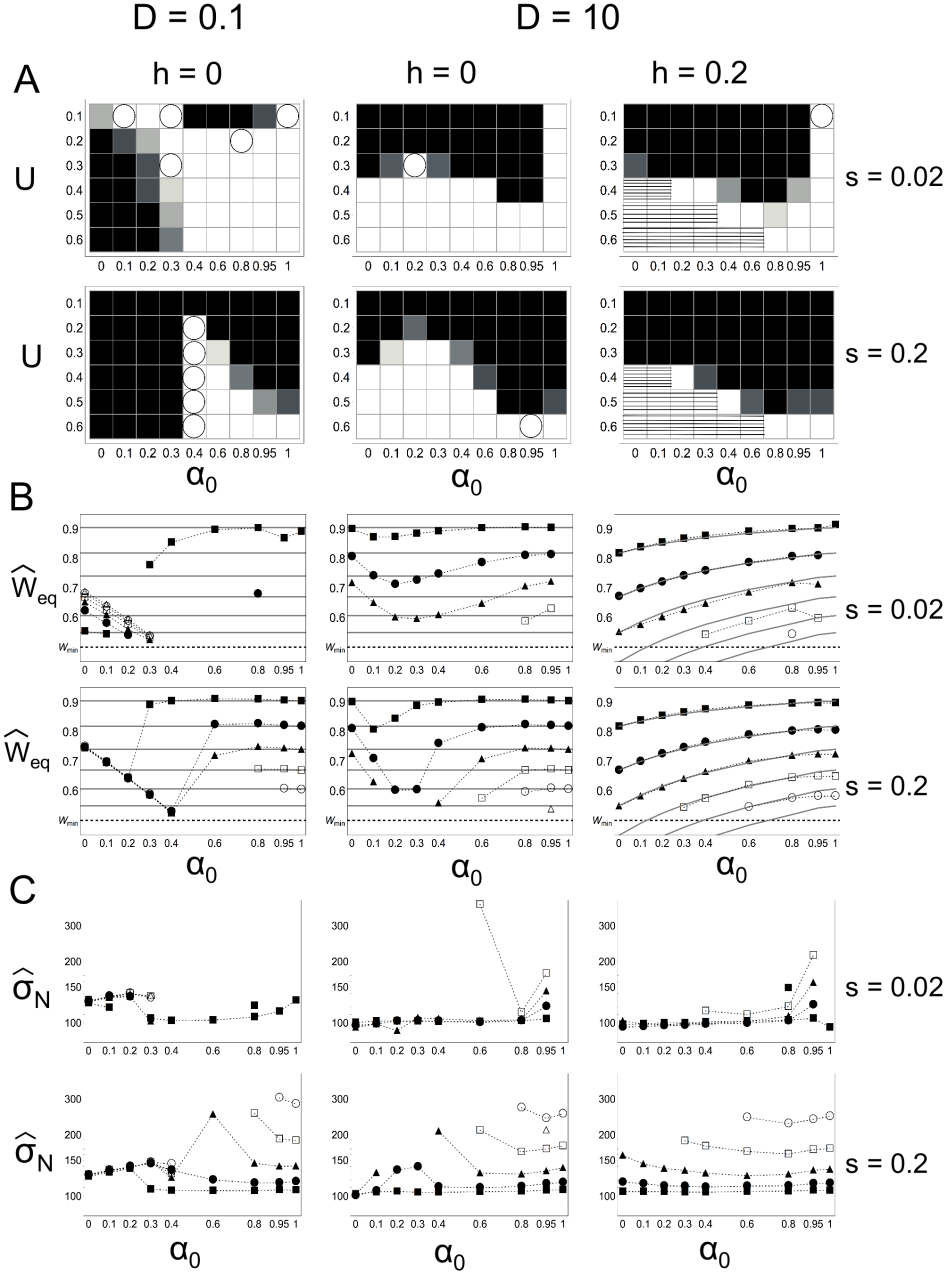
Population extinction and equilibrium. **A**) Probability of population extinction calculated from the 1000 simulations run (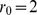 and 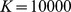), from black (0% extinction) to white (100% extinction), as a function of 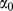. The circles indicate that more than 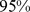 of populations went extinct. The horizontal lines indicate that deterministic extinction was predicted (Equation 3). **B**) Mean values of the observed population fitness at equilibrium across simulations 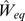 of viable populations as a function of 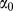 for 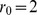 and 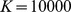. The grey lines represent the expected mean fitness 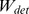 for (from top to bottom) 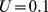, 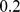, 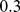, 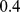, 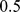 and 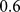. Top row 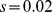 and below 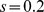. Missing points indicate parameter values for which none of the 1000 simulations run were viable. **C**) Standard deviation of population size over time at population equilibrium across simulations 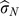 with a logarithmic scale as a function of 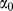, with 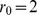 and 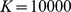. Note that this variable is underestimated for parameter sets with less than 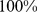 population survival, as the standard deviation of extinct populations is not taken into account. For **B** and **C**: ▪ 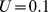, 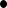 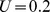, ▴ 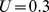, 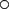 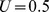, 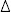 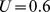.

As expected from deterministic approximations (see section “Deterministic model and expectations”, Equation 6), when mutations are only slightly linked (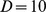) we find that for a particular intrinsic reproductive rate 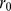, increasing values of the haploid mutation rate 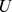 leads to higher probabilities of extinction (Figure 2A) and lower 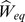 (Figure 2B). Contrary to deterministic expectations, the coefficient of selection 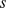 affects both population extinction and mean relative fitness. The effect of the coefficient of the selection and the proportion of selfed offspring both depend greatly on the dominance 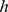 of the deleterious mutations. Generally, increasing the coefficient of selection decreases the probability of extinction at higher mutation rates and increases mean relative fitness 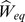.

From the deterministic equations, the proportion of selfed offspring should not affect either of these variables when mutations are completely recessive and almost recessive (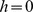). However, self-fertilisation has a non-monotonic effect on both of these variables. Between 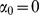 and 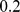 the probability of extinction increases, while 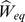 decreases. For 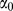 between 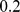 and 0.95, the opposite tendency is generally observed, the greater 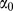, the lower the probability of extinction and the higher 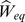. When a strictly selfing regime is imposed, no simulated populations survive when mutations are almost neutral (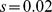) and when mutations are mildly deleterious (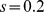) the probability of extinction (respectively the mean relative fitness) is the same, or slightly higher (respectively lower), as what is observed for 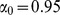. The same patterns are observed when mutations are almost recessive (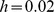). When mutations are moderately recessive (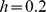), we find that the deterministic expectations are more accurate. There is generally a monotonic effect of 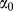 on both the probability of extinction viability and 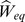, the former decreases and the latter increases with increasing 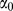. At higher mutation rates, we observe that the probability of extinction increases and 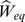 decreases at very high values of 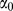 (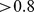). Once again, when mutations are almost neutral (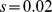), no populations survive when 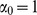. For all values of 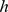, increasing 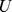 accentuates the effect of 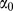 on the decrease of 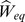 and increase of the probability of extinction.

At lower recombination rates (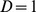 and 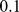), 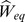 is generally not affected by 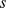 and 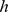, but the probability of population extinction is generally greater with increasing 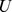 than what is observed for high recombination rates (for 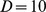). This is especially true for lower proportions of selfed offspring. The effect of recombination on viability decreases with increasing selection. We observe one particular case, when mutations are very tightly linked (the recombination rate 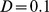), of small effect (the coefficient of selection 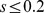) and completely recessive (dominance 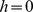), where increasing the haploid mutation rate 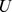 can, for low rates of 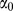, decrease the probability of extinction and increase the mean relative fitness or have no effect on either (Figures 2A and 2B). This could be due to more efficient selection against deleterious mutations at higher mutation rates, as increasing the mutation rate could increase the probability that tightly linked groups of recessive mutations are found at a homozygote state and eliminated.

As mentioned above, population fitness and demographic stochasticity alone can not fully explain population viability. Figure 2C represents the mean standard deviation of population size over time at equilibrium conditional to population survival 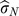. We observe that, when considering parameter sets with the same mutation rate 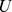, 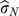 increases with increasing probability of population extinction. It is important to note that 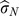 is most probably underestimated for parameter sets for which not all populations are viable, as the standard deviation of extinct populations is not taken into account during its calculation.

### Accumulation and fixation of deleterious mutations

**Figure 3:**
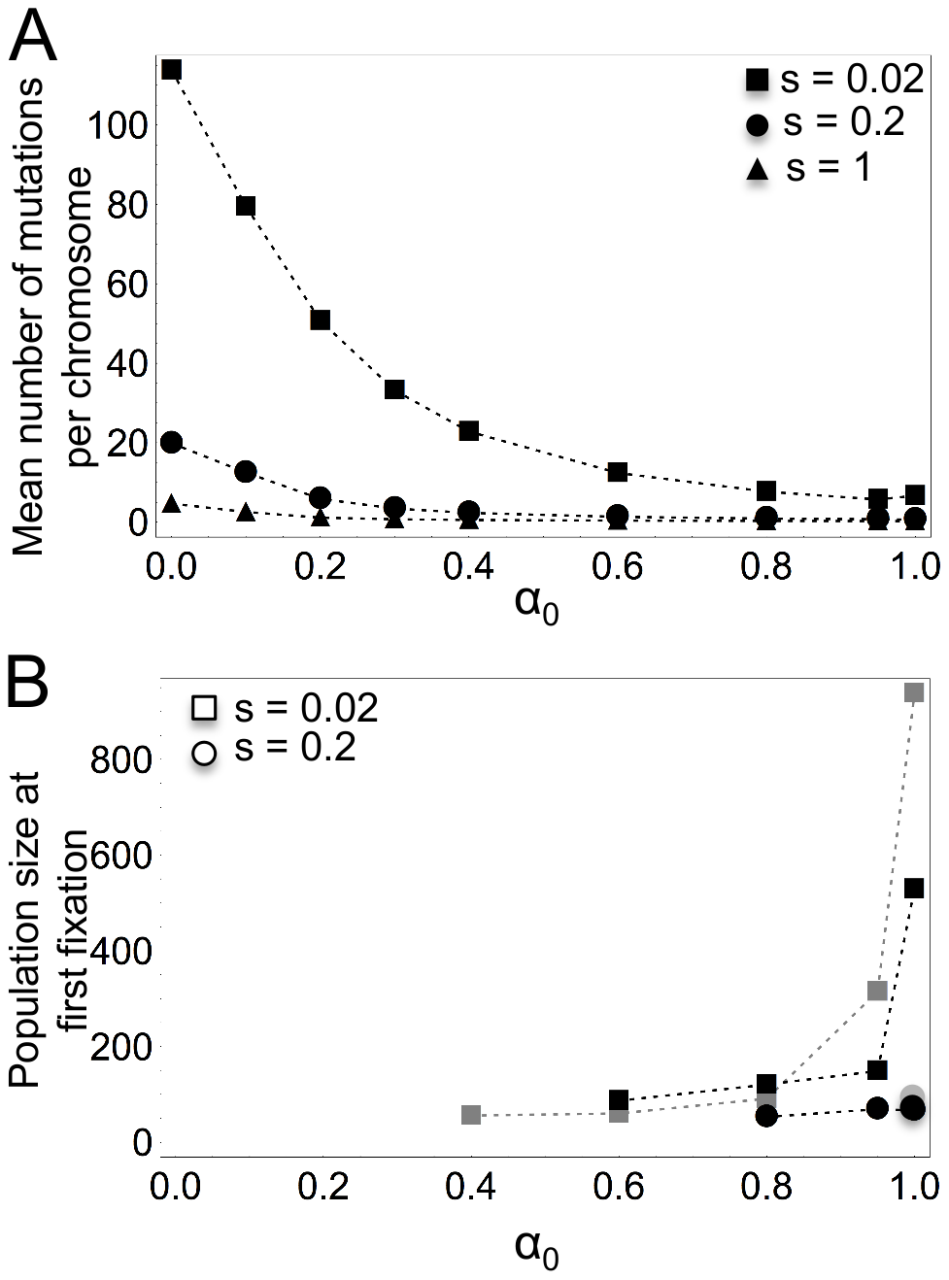
Accumulation and fixation of deleterious mutations. **A**) Mean number of mutations per chromosome equilibrium across simulations as a function of 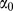. 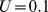, 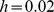, 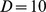 and 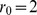. Missing points present parameter values for which no populations were viable. **B**) Mean population size at first fixation of deleterious mutations for populations greater than 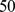 individuals as a function of 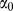. 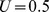 in grey, 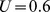 in black. 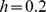, 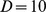 and 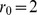.

Our results show that when deleterious mutations are fixed, it is only in populations that are very small, in other words already on their way to extinction. Even though the mean number of mutations is smaller at higher proportions of selfed offspring (Figure 3A), when populations are not viable, increasing 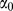 leads to the fixation of deleterious mutations at greater population sizes (Figure 3B) and with a higher probability (results not shown). The smaller the coefficient of selection and the greater the mutation rate, the greater the population size at first fixation. Therefore, if mutations are indeed fixed, they are fixed at larger population sizes when they are mildly deleterious, frequently introduced and in populations with greater proportions of selfed offspring. Mean population size at first fixation is generally relatively small compared to the carrying capacity 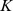 (see Figure 3B), except for one group of parameter values (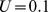, 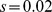, 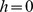 and 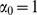, results not shown) where fixation can occur at a population size of 8000 individuals. Mean population size at first fixation is greater for high-intermediate values of 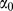 when there is little recombination than when recombination rates (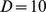) are high, whereas outcrossing populations are not affected.

The mean number of mutations per chromosome at population equilibrium across all simulations (conditional to population survival) decreases with increasing coefficients of selection 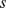 and/or the dominance 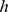 and for increasing proportions of male gametes available for selfing 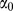 (Figure 3A shows typical results). Increasing the haploid mutation rate 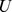 increases the mean number of mutations per chromosome. The lower number of mutations per chromosome for mutations with stronger effects (either at the homo- or heterozygous state) and for higher proportions of selfed offspring can be explained by a more efficient purging [26].

Lower recombination rates generally do not affect the mean number of mutations per chromosome, except when mutations are completely recessive (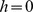) and there is almost no recombination (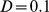). In this case the mean number of mutations per chromosome can be more than doubled for low values of 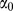 independently of the coefficient of selection 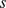, but not greatly changed for higher values of 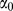 when selection is strong 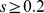.

### Mutational meltdown and time to extinction

**Figure 4:**
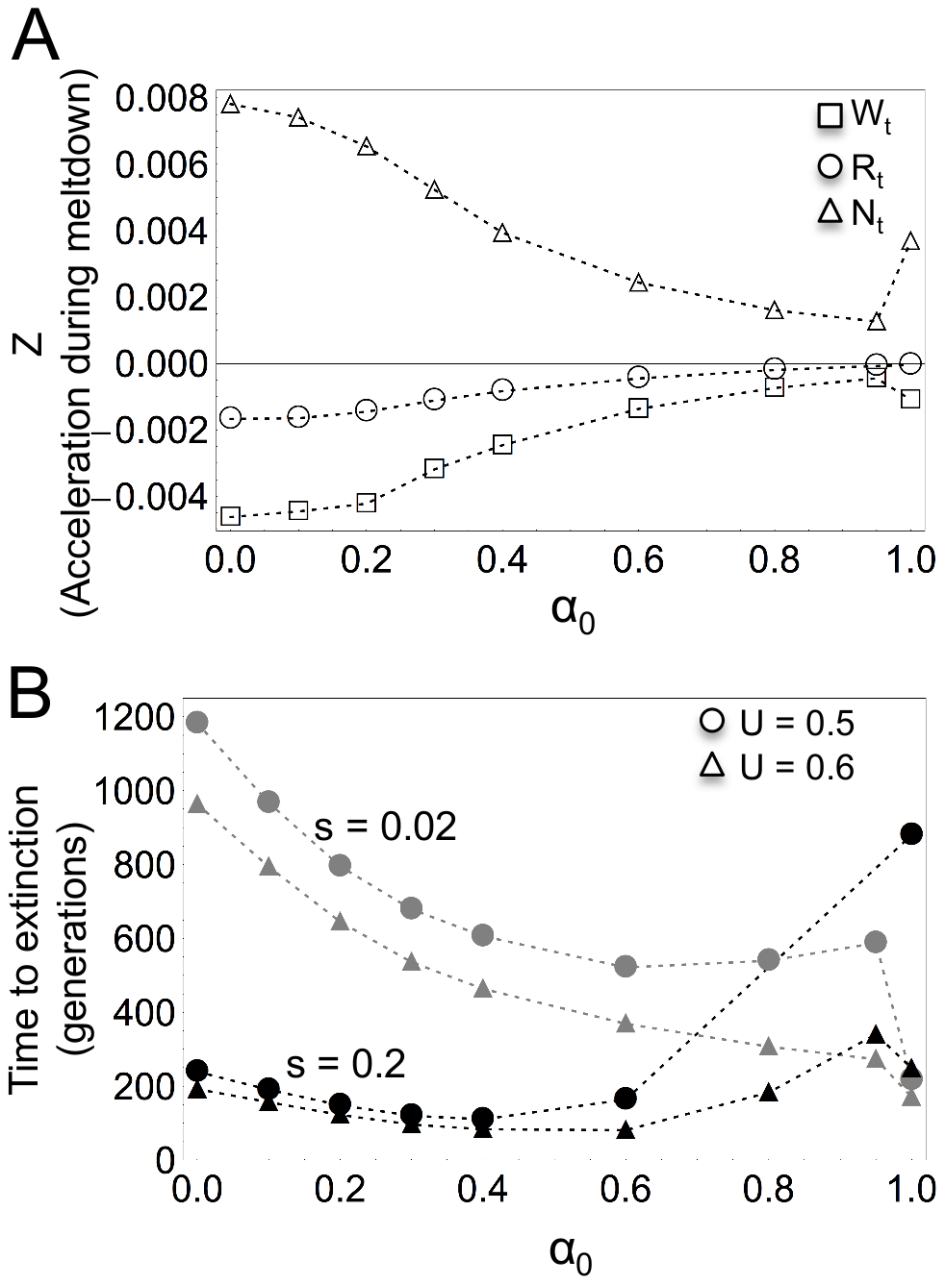
Mutational meltdown and time to extinction. **A**) Median value of acceleration in the rate of decrease of 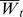, 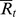 and 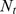, noted 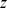, for non-viable populations from 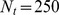 until extinction as a function of 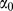. 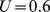, 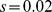, 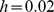, 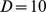, 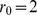 and 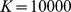. **B**) Mean time to extinction as a function of 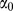. 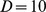, 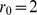 and 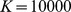.

When a population is on its way to extinction, there is a weak but significant acceleration in the decrease of the mean relative fitness 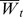 and in the mean reproductive rate 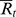, but there is a deceleration in the decrease of population size 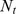 for high recombination rates (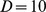, see Figure 4A). Higher mutation rates 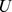, lower dominance of mutations 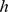 and lower proportions of selfed offspring 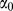 contribute to the acceleration (respectively the deceleration) of the decrease of 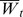 and 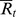 (respectively 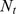). Even though both the mean relative fitness and the reproductive rate show an overall tendency to decrease at an accelerating rate, the low population density allows for the deceleration of the decrease of population size as the smaller the population size, the more resources available per individual for reproduction, as 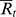 is density dependent (Equation 2).At low recombination rates, there is neither an acceleration or a deceleration in the decrease of 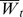, 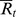 and 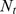.

The mean time to extinction for non-viable populations has a complex relationship with the proportion of male gametes available for selfing 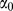 (Figure 4B). The general patterns that are observed, however, are that the effect of 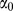 on the time to extinction is attenuated with increasing values of 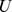. Increasing selection (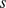 and 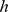) reduces the mean time to extinction for populations with low proportions of offspring produced via self-fertilisation, but increases it for higher 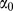 (Figure 4B). The contrary is observed when mutations are very mildly deleterious (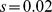) and recessive (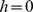 and 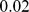), with longer times to extinction for outcrossing populations than for selfing populations. The pattern of the effect of 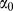 and selection on time to extinction remains unchanged when recombination rates are low.

## Discussion

It is generally accepted that selection is less effective in small populations, which could lead to their extinction due to mutational meltdown [2]–[5], whereas large populations are able to purge recurrent deleterious mutations and remain at mutation-selection balance [15], [39]. Our results suggest that there are values of genetic parameters for which even large populations cannot purge deleterious mutations fast enough to reach mutation-selection balance and go to extinction due to the increasing number of segregating mutations, which in turn increase the mutational load. This implies two things: 1) Mutation selection balance is not attainable for all genetic parameters as hypothesized by population genetics models and 2) Populations can go to rapid extinction due to segregating deleterious mutations. Self-fertilisation, while expected to allow for more efficient purging of deleterious mutations [26], does not always allow for lower probability of extinction. Population fitness as well as the amplitude of the fluctuation of population size both contribute to the eventual fate of a population, with lower population fitness and greater fluctuations leading to higher probabilities of extinction. More specifically, our results show that there is a synergistic interaction between genetics and demography, which increases the stochastic fluctuations of population size.

Empirical estimations of the genetic parameters used in our model are now becoming available. The idea that there is a strong correlation between 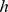 and 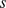 has often been shown in empirical works ([40]–[42]; also see [43]). As the dominance coefficient has been estimated as being around 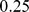 [44] and most of the deleterious mutations that make up an individual's mutational load are of small effect, this implies that the most realistic parameters we have run are for 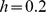 and 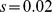. However, a new approach using a phenotypic landscape model has shed doubt on this interpretation; the dominance and the coefficient of selection of mutations might well be independent of one another [44]. This justifies our choice to study several values and combinations of these values of 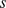 and 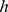 values. We have chosen to consider mutations with constant and only deleterious effects, which is one of the limits of this model. Mutations found in natural populations have variable coefficients of selection 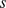 and dominance 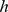 and the distributions of these variables are still under debate as they can vary not only between species [45], but also between populations [46]. How the variability of mutation affects the evolution of populations is still unclear and yet to be fully taken into account in theoretical models.

The genomic mutation rates (equivalent to 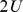 in our model), estimated empirically in various organisms range from 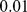 to 1 [19] and even greater [17] in eukaryotes. We therefore explore realistic mutation rates, even though in our case we consider that all new mutations are deleterious.

### Population size, viability and the mutational meltdown

Generally, if we are able to predict demographic factors (such as the intrinsic reproductive rate, the carrying capacity and density dependence) and the mean relative fitness of populations (or the mutational load), we are able to predict population size and a threshold value of mean relative fitness, below which a population is not viable (Figure 1A). However, predicting a relatively large (or non-null) population size is not enough to guarantee population survival within a relatively long time scale. In our model we find that population extinction is not due to demographic stochasticity alone, but to increased levels of stochasticity that result from the interaction between demography and genetics.

The importance of this interaction on population extinction has already been suggested in literature [47]. Our results find that it is indeed non-negligible as shown by the fluctuation of population size over time in our model, which is a result of demographic stochasticity and a dynamic mutation load or relative fitness, which are not independent of one another (Figure 1D). The mean fluctuation of the population size for a given set of parameter values (open circles) are too great to be due to demographic stochasticity alone (full circles). Even though stochastic processes affect the reproductive rate, contemporary stochasticity alone does not account for this variance. Past stochastic events, or the mutational history of the population (where the deleterious mutations are in the genome, and at what frequencies), can also influence this variable, as observed in Figure 1D, where the standard deviation of population size 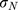 (grey points) varies greatly around the mean standard deviation observed 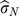 (open circles). For parameter values where the probability of extinction is different than 0 and 1, the importance of past stochastic events, is even more evident as the fate of a population is not sealed. The importance of past mutational events has also been observed in experimental mutation accumulation lines, where replicate populations with the same genetic origin do not all go to extinction (e.g. for yeast populations [8]). We propose, that in order to predict the probability of extinction of populations, it is not only important to predict the mean fitness, but also the fluctuation of population size. Further exploration of this model is required to estimate how the genetic and demographic parameters affect the amplitude of the fluctuations of population size. No theoretical work, to our knowledge, has taken on this question from a demo-genetic point of view.

Clarke's [23] work highlights the importance of the effect of selection on demography, and his prediction that taking into account the accumulation of deleterious mutations throughout the genome would allow a significant decrease of population size is confirmed by our model, where in some cases populations go to extinction. It has been suggested that the timing of selection is crucial in order to assess the effect of the mutation load on population size [17], [23]. In our model we have chosen relative fitness to affect only the reproductive rate. However, it is possible that selection that affects the consumption of resources (*K*) could lead to different results [23]. It is often considered that selection has no effect on demography (e.g [17]), but if the mutation load has a direct effect on an individual's reproductive capacity, as is the case in our model, then the effect of selection on population size cannot be ignored. This has important implications on how data on population size from natural populations should be interpreted (see below).

The importance of the reproductive rate concerning population extinction has already been suggested by other models, where populations with higher intrinsic reproductive rates have longer times to extinction [4], [48] or lower probabilities of extinction [14]. These predictions are an inherent property of our model, as populations with high intrinsic reproductive rates *r*_0_ are expected to be viable at higher mutational loads (Equation 6), and are supported by our stochastic simulations (Figure 1B). In our model, we consider a stable environment, which is an unrealistic hypothesis. It is therefore probable that we overestimate population viability, as shown by Higgins and Lynch's model [49], which, upon taking environmental stochasticity into account, suggests that it increases the rate of accumulation of deleterious mutations.

To what extent are we capable of estimating the mean relative fitness (or the mutational load)? In spite of explicitly considering demography, we find that the simplified deterministic predictions of the mutation load are reliable when mutations have a strong effect (*s* = 0.2 and *h* = 0.2). However, when mutations are almost neutral, mean fitness is overestimated, especially when the mutations are recessive (*h* = 0) and the genomic recombination rate is low as the number of mutations per chromosome is increased [50], affecting the purging process. The deterministic expectations of mean fitness when comparing them to simulated results have already been shown to be reliable by population genetics models [21], [22], however none of these models explicitly include the effect of demography. This interaction, between genetics and demography, could in fact be important, as shown by the unexpected non-monotonic relationship between population fitness and the proportion of selfed offspring in our model (Figure 2B), which we discuss below.

It is important to note that a high mutation rate and a large number of segregating deleterious mutations do not necessarily lead to a higher mutation load. In the case of very little recombination (*D* = 0.1), increasing the mutation rate increases mean fitness when mutations are recessive and of small effect, and hence decreases the mutation load. Therefore, the mutation rate in itself is not sufficient, and depending on the effects of deleterious mutations and the recombination rates, increasing the genomic mutation rate does not always lead to an increase in the mutational load. The extreme case of very little recombination could also be translated as the existence of genomic regions with low recombination rates known as cold-spots (reviewed in [51]), allowing for the accumulation of deleterious mutations ([52], p. 555). The existence of such genomic regions could in fact have an important influence on the survival of populations. Low rates of recombination are expected to reduce population fitness and increase the rate of fixation of deleterious mutations [53]. Though our results confirm this for mutations that are moderately recessive (*h* = 0.2), it is not the case when considering recessive mutations, where the contrary is observed. At such low recombination rates, the high levels of linkage-disequilibrium lead to the formation of tightly linked groups of mutations. These groups of mutations act as a single “super locus”. When the mutations are recessive, this load remains silent at the heterozygous state, but once at the homozygous state, the multiplicative effects of these small mutations are expressed and lead to a very deleterious effect. The relative fitness of individuals that become homozygous for only one of these different super loci is extremely low. In this case, the advantage of outcrossing is much higher, as outcrossed offspring have a higher probability of being heterozygous at these loci than selfed offspring.

From our simulated results, we conclude that when populations are on their way to extinction, whether we observe a mutational meltdown depends not only on the values of the genetic parameters, but also on the variable measured. Due to the nature of the density dependence in this model, the decrease in population size decelerates when population density is low: the smaller the population size, the more resources available to the few remaining individuals. Even though both the reproductive rate and the mean relative fitness do show an acceleration in their decrease when populations are on their way to extinction, we find that the existence of this mutational meltdown depends greatly on the effect of the deleterious mutations, the mutation rate, and the proportion of offspring produced via self-fertilisation.

The importance of segregating mutations has already been suggested by Lynch *et al.* [4], but this work concluded that fixation had a greater effect on the meltdown. Our model suggests that the fixation of deleterious mutations is a consequence rather than a cause of decline towards population extinction, this however could be due to the fact that in Lynch *et al.*'s [4] model the genetic load affected offspring survival, whereas in our model there is a direct link between the reproductive rate and the mutational load. The effect of the accumulation of segregating deleterious mutations has been considered to be negligible when considering large populations [15], [16], even more so when considering their eventual extinction over a short time scale because of this process (but see [14]). This does not seem to be the case when considering the interaction between demography and genetics. A feed-back loop between these two properties seems to lead to a “cost of purging”: Unfit individuals do not reproduce, decreasing population size at the next generation, which in turn increases the effect of drift, leading to a lower efficiency of purging and more unfit individuals. This continual increase of the number of segregating deleterious mutations eventually leads to a demo-genetic extinction. Mutation-selection balance is therefore not the automatic fate of initially large populations, and the cost of purging can lead to a very rapid extinction [14].

### How does selfing affect population size and viability?

Our results indicate that selfing has an effect both on population size and viability. We often observe that selfing populations have lower probability of extinction than outcrossing populations at higher mutation rates (see Figure 2A), especially when there is strong selection, in which case selfers are always expected to have larger population sizes. However, when selection is weak, we find that strict-selfing and low levels of selfing (but not strictly outcrossing) hinder both size and viability. As this has not been noted in other models, even when genetic drift is taken into account [21], [36], it is possibly a consequence of the interaction between genetics and demography. A possible hypothesis to explain this observation is that the viability of populations concerning the accumulation of deleterious mutations depends on two opposing properties 1) The purge of these mutations and 2) The avoidance of expressing them. Outcrossers accumulate deleterious mutations [26], but avoid the cost of inbreeding depression [54], with most of their mutations being at a heterozygous state. Selfers purge these mutations [26] and even though they do not accumulate as many as outcrossers do, many are at a homozygous state [55]. We therefore propose that populations with low proportions of offspring produced via self-fertilisation suffer from both the inconveniences of outcrossers and selfers, not only do they accumulate deleterious mutations, as purging is not as efficient as for high proportions of selfed offspring, they also express them, and suffer from the demographic cost of purging.

The effect of self-fertilisation on the extinction of populations due to the accumulation of deleterious mutations has long been debated [4], [28], [56]. Our results suggest that the accumulation, hence fixation, of deleterious mutations is probably an insufficient explanation for higher extinction rates. In spite of this model's limitations, we find that even though self-fertilisation does affect population extinction due to genetic deterioration, the effects of the genetic parameters are complex and do not result in a simple pattern of the effect of selfing on the time to extinction. When selection is weak, strict outcrossers are less likely to go extinct than strict selfers, agreeing with Lynch *et al.*'s [4] results. However, allowing for a small amount of outcrossing (e.g. a proportion of male gametes available for selfing *α*_0_ = 0.95) is enough to greatly decrease the probability of extinction, even allowing for a higher probability of population survival at higher mutation rates than for strict outcrossing (see Figure 2A). Strong selection reverses this observation, with strict outcrossers being more prone to a mutational meltdown than selfers, in accordance with Bernardes' [14] results. What rate of selfing is more likely to cause extinction or lead to population vulnerability is not clear and greatly depends on both the genetic (mutation rates, genomic recombination rates, deleterious effects of mutations) and demographic (intrinsic reproductive rate) parameters. It has already been suggested that selfing has a greater effect on population extinction when considering the fate of beneficial mutations and their effects after environmental change. For instance Glémin and Ronfort [57] showed that if adaptation is due to standing variation, then outcrossers are less prone to extinction than selfers. Their model, however, is not demographically explicit.

When considering genomic cold-spots with low recombination rates, outcrossers are greatly advantaged when mutations are recessive, as they do not express these accumulated mutations. The lower viability of selfing populations in our results for such low recombination rates supports the observation that selfing species could be more likely to evolve higher recombination rates, in order to avoid the hitchhiking of deleterious mutations [58]. The difference in extinction rates between outcrossers and selfers observed empirically [29] could perhaps be due to such genomic regions where mutations of small effect segregate.

The non-monotonic effect of selfing on the probability of extinction in our results, could offer a possible explanation to the differences in extinction rates between selfers and outcrossers within the same family. For a transition to be successful, the transition in the reproductive mode has to be of large effect, going from complete outcrossing to high proportions of selfed offspring, for in some cases the mutational load that an outcrossing population could put up with could prove lethal for a reproductive mode with low proportions of offspring produced via self-fertilisation. The observed high extinction rates could therefore be related to the transition process and not the reproductive mode in itself.

### Empirical implications

How can the correlation between population size and population fitness be interpreted? In most empirical works, a positive correlation between the two is translated as the negative effect of a small population size on population fitness due to inbreeding, the fixation of deleterious mutations or a lack of reproductive assurance (for example [59], [60]). Another possible interpretation which is not often considered is simply that population size is a consequence and not the initial cause of a high mutation load, just as in some cases small population size does not seem to lead to a decline in fitness [61].

Generally, small populations are considered to be most at risk of extinction within a relatively short time frame due to inbreeding depression, mutational meltdown and demographic stochasticity [1], [15], [47]. Empirical experiments have therefore concentrated on the extinction of small populations, through the accumulation of deleterious mutations [6]–[10]. Even though the fixation of deleterious mutations can lead to the mutational meltdown of small populations [3]–[5], our results suggest that the interaction between demography and genetics can lead to the extinction in large populations due to segregating mutations alone and at relatively fast rates. In initially large populations, once the “mutational meltdown” is underway, fixation is rare and is a consequence rather than a cause of population decline. The importance of segregating mutations in population decline could have implications in conservation biology, as in most empirical studies, it is automatically assumed that the load leading to population decline is fixed or almost fixed [62], [63]. If population decline is mostly due to segregating mutations, then there exists a real potential of purging the deleterious load through conservation efforts. Small populations are also expected to be more prone to demographic stochasticity, which should act more rapidly on population extinction than genetic factors [1], [47], as has been shown through empirical experiments [64]. The higher levels of stochasticity in the variation of population size observed due to this interaction compared to the expected effect of demographic stochasticity alone in our results (Figure 1D) indicate that stochastic events (that are not due to external factors such as environmental stochasticity) are not only detrimental in small populations, but can also be so in large populations.

Our results suggest that measuring the decline in population size could be misleading when attempting to asses whether a population is going into a mutational meltdown or not. Depending on the density dependence, a population on the way to extinction could seem to be demographically stable, as the decrease of population size could potentially decelerate with time, becoming barely detectable. As the mutational load is not accessible, measuring the acceleration of the decline of the mean relative fitness is not possible in natural populations. However, measuring the acceleration in the decline of the mean reproductive rate over several generations could be a more informative measure concerning population extinction, or the mutational meltdown, and is empirically more accessible. The lack or rarity of a mutational vortex when our simulated populations are on the decline could indicate that, if the segregating deleterious mutations can be purged at any time, then, as there is very little or no increase in the rate of reduction of population size, conservation efforts could be applied successfully even when populations reach relatively small sizes.

In conservation biology, Population Viability Analyses (PVA) are the most frequently used tool for estimating the probability of population extinction. PVAs take mostly demographic data and parameters into consideration and do not take into account genetics explicitly. They have proved useful and accurate when considering external pressures (i.e. over-fishing, fragmentation, etc.) that affect population demography. However, the effectiveness of PVA's remains ambiguous, as even though they can be relatively accurate predictors of the evolution of population demography[65], in other cases the population growth rates can be over-estimated [66]. Could the overestimation of growth rates of growth rates be due to the omission of the genetic effects on population demography?

The direct relationship between higher intrinsic reproductive rates and greater population sizes for the same relative fitness in our model (Equation 3, Figure 1B) leads to a relationship between the reproductive and mutational rates in viable populations (Equation 6). This could mean that for a population with a high genomic mutation rate to be viable, it must have a large enough intrinsic reproductive rate. This relationship has not been studied either theoretically or empirically, and it could be interesting to perform a comparative analysis between species to test if there is such a relationship. We suggest that there could be a correlation between the genomic mutation rates of a species and the number of gametes produced, which could represent the intrinsic reproductive capacity.
