# Correction: The Interaction between Selection, Demography and Selfing and How It Affects Population Viability

**DOI:** 10.1371/journal.pone.0091627

**Published:** 2014-02-28

**Authors:** The PLOS ONE Staff

The name of the first author is incorrectly represented in the Citation and Copyright. The correct Citation is: Abu Awad D, Gallina S, Bonamy C, Billiard S (2014) The Interaction between Selection, Demography and Selfing and How It Affects Population Viability. PLoS ONE 9(1):e86125. doi:10.1371/journal.pone.0086125.

The Copyright statement should read: "© 2014 Abu Awad et al. This is an open-access article distributed under the terms of the Creative Commons Attribution License, which permits unrestricted use, distribution, and reproduction in any medium, provided the original author and source are credited."
